# The Challenge of Cross-Cultural Care Encounters: Perspective of Imported Nurses in Lhasa, Tibet

**DOI:** 10.1155/2020/3159178

**Published:** 2020-04-08

**Authors:** Mu Bai, Xin Sui, Changli Zhou, Yuewei Li, Jinwei Li, Ruitong Gao, Zhen Du, Linqi Xu, Feng Li

**Affiliations:** ^1^School of Nursing, Jilin University, 965 Xinjiang Street, Changchun, Jilin 130021, China; ^2^Department of Respiratory Medicine and Gastroenterology, Tibet Autonomous Region Second People's Hospital, Lhasa 850000, Tibet Autonomous Region, China

## Abstract

**Aims:**

The purpose of this study was to describe the challenge of cross-cultural care encounters from perspective of imported nurses in Lhasa, Tibet, as well as investigate the relationship of cross-cultural care encounters and its influencing factors.

**Methods:**

A cross-sectional survey was designed among 300 imported nurses and 255 patients selected from four comprehensive hospitals (including two Grade III Class A hospitals and two Grade III Class B hospitals) in Lhasa. The average number, standard deviations, constituent ratios, *T*-tests, rank-sum tests, one-way ANOVAs, multiple stepwise regression analyses, and Pearson correlation analysis were used to analyze cross-cultural care encounters and its influencing factors. *P* < 0.05 was considered statistically significant.

**Results:**

The cross-cultural care encounter of nurses was 61.73 ± 11.86, mainly relating to age, technical titles, Tibetan language ability, and participation in humanistic training. Age, gender, educational level, technical titles, Tibetan language ability, years working in Tibet, and participation in language and humanities training were the influencing factors (*P* < 0.05). The average total score of culturally competent care of imported nurses in Lhasa was 218 ± 31.09. Cross-cultural care encounters of nurses were positively correlated with culturally competent care (*r* = 0.126, *P* < 0.01) and the needs of patients' cultural care (*r* = 0.183).

**Conclusion:**

The scores of culturally competent care and cross-cultural care encounter of imported nurses were at a high level, and their culturally competent care was in the second stage of “conscious and incapable” status. The cross-cultural care encounter of nurses is positively related to culturally competent care and the needs of patients' cultural care. Abilities of language communication, understanding of Tibetan culture, and enhancement of the cultural ability needed optimization.

## 1. Introduction

### 1.1. Background

Tibet is in the southwestern border of China and is an underdeveloped area compared with the rest of the country. Due to its unique geographical location and historical background, medical advances are backward and unevenly distributed [1–3]. Thanks to national policies, major hospitals in Tibet have introduced health professionals from mainland China, and the number of health professionals has continued to increase [4, 5]. Tibet is also an international tourist destination that attracts tourists from many countries. Due to different cultural backgrounds, Tibet has formed a multicultural status.

Encounter was often used as a term in caring science, indexing a human-to-human encounter, and was also depicted as a relationship between care providers and patients [6, 7]. A cross-cultural care encounter refers to the cultural competence and experience of the contact between care providers and patients from different cultural backgrounds [8, 9], including communication barriers between nurses and patients, eating habits, cross-cultural competency, etiquette, and traditional habits.

To improve the quality of care in Tibetan hospitals, hospitals have introduced many nursing staff from mainland China. However, in the nursing practice, cross-cultural care differences often arose between nurses and patients due to different language and cultural backgrounds [10], which affected both sides. The patients understand the nurses' words and deeds from a Tibetan cultural perspective, and the nurses do not understand the patients' thoughts and intentions [11]. Therefore, both sides misinterpret different views on care, and patients might have rejection behaviours, which could damage the relationship between nurses and patients, leading to misunderstanding of care measures, deliberate disobedience, nonacceptance, or low care quality satisfaction rate. The imported nurses could misinterpret patients' tendencies without forming awareness, sensitivity, and basic knowledge of Tibetan culture. With the widespread introduction of nursing staff, imported nurses in general as well as cross-cultural care are challenged increasingly by cross-cultural care encounters resulting from various ethnic backgrounds.

In order to increase better reception by patients and improve the nursing service patient satisfaction rate [9], we would better further optimize the current status of the cross-cultural care encounter between imported nurses and patients.

Based on Leininger's Transcultural Nursing Theory, when caregivers provide culturally consistent care, the cross-cultural care encounter can be optimized [12]. Studies highlighted that the cross-cultural care encounter was associated with providing culturally competent care [13] and effective communication with patients from multicultural and ethnic backgrounds who own different beliefs, practices, values, and languages [9, 14–16]. Different language and social and cultural barriers between healthcare providers and patients are some of the challenges for cross-cultural care encounters [17].

Therefore, according to the current status of the cross-cultural care encounter, we wonder what the main influencing factors in Lhasa are. In addition, we want to know if the cross-cultural care encounter in Lhasa is influenced by culturally competent care and the cultural care needs of patients and what the relationship among the three factors is.

### 1.2. Objectives

Few articles have tried to study the cultural situation of Lhasa. No researches had shown the relationship of the current status of the cross-cultural care encounter, culturally competent care, and the cultural care needs of patients. This study is aimed at providing a theoretical basis for Tibet nursing staff to deliver cross-cultural care better, optimizing cross-cultural care encounters, and improving the quality of nursing care in Lhasa, the largest medical care center in Tibet, which was selected to investigate the current status of the cross-cultural care encounter, culturally competent care, and the cultural care needs of patients; explore the relationship among these three factors; and propose corresponding intervention measures.

## 2. Methods

### 2.1. Respondents

The study was a cross-sectional study from July to November 2017 that used data from four comprehensive hospitals (including two Grade III Class A hospitals and two Grade III Class B hospitals) in Lhasa, Tibet. In this study, we used a convenience-based sampling survey on imported nurses and Tibetan patients who met the inclusion and exclusion criteria and who were willing to participate following the provision of informed consent.

The inclusion criteria for nurses were the following: on-the-job nurses with adequate professional qualifications; imported nurses from mainland China, rather than Tibetan students; non-Tibetan nurses who cannot communicate effectively in the Tibetan language; and provision of informed consent to participate in this investigation. The exclusion criteria for nurses were the following: mixed nurses from Tibet and Han ethnicities, nurses not based in hospitals, trainee nurses, and advanced students. The survey tools used were the general data questionnaire, culturally competent care scale, and cross-cultural care encounter questionnaire.

The patient inclusion criteria were Tibetan ≥ 18 years and provision of informed consent to participate in this investigation. The patient exclusion criteria were mixed patients from Tibet and Han ethnicities, patients with mental illness or severe cardiovascular disease, and patients with cognitive disorders or with difficulty filling the questionnaire. Willingness to participate in this study required the completion of a general information questionnaire and cross-cultural care need questionnaire.

In total, 300 imported nurses and 255 Tibetan patients were included in the study.

### 2.2. Questionnaires

#### 2.2.1. General Information Questionnaire

This questionnaire had two parts: personal and social data. Personal data included age, gender, nationality, and marital status. Social data included the educational level, whether cultural and Tibetan language training was received, years working in Tibet, job title, the technology used at work, department, and hospital level.

#### 2.2.2. Cultural Competence Health Practitioner Assessment (CCHPA)

CCHPA was developed by the National Cultural Care Center of the United States as a tool for measuring the cultural care abilities of health practitioners. The assessment incorporates three large dimensions and seven subdimensions [18]. Cronbach's score coefficient was 0.932, with good reliability and validity.

#### 2.2.3. Cross-Cultural Care Encounter Questionnaire

This questionnaire was used to investigate cross-cultural situations experienced by imported nurses during their clinical work in Lhasa. The final version of this questionnaire was generated after the revision of the Problems in the Multicultural Nursing Care for Foreign Patients scale developed by Peng et al. [19]. Using the Delphi method, six Tibetan and two Han Chinese nursing experts were invited to review and evaluate the questionnaire content. The dimensions and items included in the final questionnaire were intended to accurately express the current status of cross-cultural care encounters experienced by imported nurses. The questionnaire content was found representative. Based on a standardized internal consistency analysis, Cronbach's *α* was 0.843, suggesting good reliability and validity. In total, the questionnaire included 20 items across 8 dimensions. The total possible score ranged from 20 to 100 points. The higher the score, the more cross-cultural nursing experience an imported nurse has.

#### 2.2.4. Cross-Cultural Care Need Questionnaire

This questionnaire was used to investigate Tibetan patients' cross-cultural nursing care needs and was based on the Investigation of the Cultural Care Need Scale of Foreign Patients by Mou [20]. A Tibetan version was created after local revision. The dimensions and items were intended to accurately express the demands among Tibetan patients for cross-cultural nursing care. The questionnaire content was found representative, with a Cronbach's *α* internal consistency coefficient of 0.898. In total, the questionnaire included 20 items across 7 dimensions. The scores ranged from 20 to 100 points. The higher the score, the more cross-cultural care needs a Tibetan patient has.

### 2.3. Data Collection

In this study, we adopted a convenience-based sampling method, and questionnaires were distributed by researchers to team members and trained nurses in the same department. In total, five investigators participated in this distribution process. All questionnaires were filled by the respondents and were subjected to on-site investigation, on-site recovery, and unified coding. A total of 320 questionnaires were issued, and 310 were recovered, but only 300 were valid questionnaires (effective rate of 96.8%). A total of 270 questionnaires were distributed to Tibetan patients, and 255 valid questionnaires were recovered (effective recovery rate of 94.4%).

### 2.4. Statistical Analyses

A database was established using the EpiData3.1 software, and data analysis was conducted using SPSS v21.0. The average number, standard deviations, and constituent ratios were used to describe general information, cross-cultural care status, and culturally competent care of nurses. *T*-tests, rank-sum tests, one-way ANOVAs, and multiple stepwise regression analyses were used to analyze the factors influencing nurses' cross-cultural care encounters. Pearson correlation analysis was used to explore the relationship between nurses' culturally competent care, cross-cultural care encounter scores, and Tibetan patients' cultural care needs. *P* < 0.05 was considered statistically significant.

## 3. Results

### 3.1. General Information of Tibetan Patients

A total of 255 patients were enrolled in the study, including 110 males (43.4%), 190 (62.7%) patients did not understand Chinese, 247 (96.9%) had religious beliefs, and 146 (57.3%) were farmers and herdsmen. Details of patients' characteristics are shown in [Supplementary-material supplementary-material-1].

### 3.2. General Information on Imported Nurses

Among the 300 Lhasa imported nurses, 284 (94.7%) were women and 16 (5.3%) were men; the ratio between them was 17.87 : 1; most nurses were 26 to 30 years old, accounting for 36.7%. The age of entry into Tibet was ≤5 years, accounting for 39.7%; the gender and age distribution were consistent with the statistical reports of domestic nursing staff [21]. Nurses were mainly undergraduate and from junior colleges. Nurses who attended the humanities nursing training accounted for 15.7%, nurses who received Tibetan language training accounted for 19.0%, nurses who use the Tibetan language to exchange account for 13.0%, and nurses who have religious beliefs account for 7%. The nursing characteristics are detailed in [Supplementary-material supplementary-material-1].

### 3.3. Patient Cross-Cultural Nursing Care Needs

The total score of cross-cultural nursing care for Tibetan patients was 60.56 ± 13.73, and the average score of each dimension was 3.03 ± 0.80 points. Details are shown in [Supplementary-material supplementary-material-1].

### 3.4. Imported Nurse Cross-Cultural Care Encounter

In this study, the scores of culturally competent care of imported nurses in Lhasa were 208.58 ± 25.09, the average score of each dimension was 3.47 ± 0.24 points, and the cross-cultural care encounter scores were 61.73 ± 11.86 points. The entries were divided into 3.09 ± 0.59 points; the scores of each dimension are shown in Supplements [Supplementary-material supplementary-material-1] and [Supplementary-material supplementary-material-1].

The results of a single-factor analysis showed that the cross-cultural care encounter scores of imported nurses were related to age, gender, marital status, educational level, title, whether they participated in Tibetan language and humanities training and Tibetan language proficiency, and other factors. These values were statistically significant (*P* < 0.05). Regression analysis showed that the main influencing factors of the cross-cultural care encounter from the imported nurses included age, title, Tibetan language proficiency, and whether they participated in humanities training. The specific results are shown in Tables 1 and 2.

The cross-cultural care encounter, culturally competent care, and scores of all dimensions are in line with the normal distribution. Therefore, Pearson correlation analysis was obtained from the total score of cross-cultural care encounters, the scores of cross-cultural care ability, and each dimension. The results showed that cross-cultural care encounters of the introduced personnel were positively correlated with culturally competent care (*r* = 0.126, *P* < 0.01). In other words, the higher the cross-cultural care ability, the more cross-cultural care encounters imported nurses had. Details are shown in Table 3.

### 3.5. Cross-Cultural Care Encounters of Nurses Were Related to Patients' Cultural Care Needs

According to the normal test analysis, the cross-cultural care encounter of nurses, the total scores of cross-cultural care needs of patients, and each dimension were in a normal distribution. Therefore, a Pearson correlation analysis was carried out. The results showed that the cross-cultural care encounter of nurses was positively correlated with the needs of patients' cultural care (*r* = 0.183). In other words, the more cultural care needs the patients had, the more cross-cultural care encounters the imported nurses could experience. Details are shown in Table 4.

## 4. Discussion

Tibet has a high altitude and cold area characterized by lacking oxygen, deficient transportation, inadequate infrastructure, shortage of medical staff, and relatively scarce medical resources with uneven distribution. People in Tibet have difficulty seeing doctors. In recent years, thanks to national policies, the number of imported medical staff has continued to rise, allowing the majority of patients to enjoy high-level medical services. However, during medical treatment, we saw a series of cross-cultural care encounters created by multicultural care.

It was known that culturally competent health service was of critical importance; however, it was presented as a frequent and common challenge for caregivers worldwide [22]. Based on Leininger's Transcultural Nursing Theory, our study revealed the cross-cultural care encounters of imported nurses in Lhasa, Tibet, and explored how Tibetan patients' cultural care needs and nurses' culturally competent care influenced it, as well as how to improve the culturally competent care of clinical nurses and optimize the quality of nursing services.

### 4.1. Characteristics of Tibetan Patients

The educational level of patients in this study was generally low, and most of them had a primary school level (41.2%), which could be related to the majority of Tibetan involvement in agricultural activities (43.2%), which is consistent with the demographic characteristics of Tibet [23]. This study also found that under different occupational activities, home areas, and other groups, patients have different Chinese proficiency, and the Chinese ability in urban areas is higher.

### 4.2. Characteristics of Imported Nurses

Most of the nursing staff imported from mainland China to Lhasa were young nurses, and there are more women than men, which follows the basic trend of skilled worker recruitment in Tibet [24]. Among the respondents, Han ethnicity nurses accounted for 98%, Hui ethnicity only accounted for 2%, and head nurses accounted for 10.3%. Only 15.7% participated in humanistic nursing training. Compared with the data of domestic scholars such as that of Hu [25], Lhasa nurses received relatively low humane care training. In this study, the number of nurses who received Tibetan language training accounted for only 19.0%, and only 13.0% of the total can communicate in the Tibetan language, which was associated with the hospital management system and did not involve training for non-Tibetan nurses in the continuous educational program.

### 4.3. Cross-Cultural Care Encounters of Imported Nurses

Nursing staff can meet the growing cultural care needs of Tibetan patients and optimize cross-cultural care encounters between nurses and patients only after gaining a substantial degree of understanding of cross-cultural nursing theory and Tibetan folk culture.

The results showed that the total score of nursing staff's cross-cultural care encounters was 61.73 ± 11.86, and that was at a high level, suggesting that Lhasa imported nurses are experiencing a cross-cultural care encounter ([Supplementary-material supplementary-material-1]).

Through single-factor and multifactor analysis (Tables 1 and 2), the influencing factors of the cross-cultural care encounters of imported nursing staff include age, technical titles, Tibetan language proficiency, and whether they participated in humanities training.

The results showed that young nurses experienced a higher cross-cultural care encounter than older nurses. Campinha-Bacote [13] showed that with the increasing age and experience of nurses, the clinical experience was richer, and the culturally competent care was also better. Some domestic studies have shown that young nurses have lower culturally competent care than middle-aged or old nurses [26, 27]. This could be related to the lack of cross-cultural nursing theory education and humane nursing training during continuing educational programs in Lhasa hospitals. Huang et al. [28] as well as Sulstarova [29] showed us that cultural competency requires specific knowledge and clinical skill, as it will contribute to shortening health disparities through assessing patients' health beliefs and cultural needs using their cultural knowledge, awareness, attitudes, and skills [30, 31].

Nurses with different professional titles experienced different cross-cultural care encounters, and the scores of nurses with intermediate titles were significantly lower than those with junior titles. The cross-cultural care encounter between nurses and patients was alleviated, which is consistent with domestic study results [32]. For many nursing schools and hospital in China, the formal inclusion of the course of “cross-cultural nursing” occurred late [33], which decrease knowledge about the concept and theory of cross-cultural nursing. Furthermore, imported nurses did not get training on the Tibetan language and Tibetan folk culture, which can also contribute to these results [11].

The results also showed that the imported nurses who can communicate in the Tibetan language have significantly reduced the cross-cultural care encounter. Consistent with some international research results, in the United States, immigrant nurses with multilingual skills have significantly reduced cross-cultural care encounters [34]. Studies in this topic have shown that the higher the English level of clinical nurses, the less cross-cultural care encounter experienced [35]. As the main source of information exchange, language is the primary barrier, which directly affects the satisfaction of care [36]. Nurses should strive to overcome the cross-cultural care encounter and eliminate the problem caused by language barriers [37].

The longer the clinical nursing practice in Tibet, the less the cross-cultural encounter nurses experienced. In cross-cultural care, optimizing cross-cultural experience is a long-term activity that requires external encouragement and training [38, 39] as well as the accumulation of personal experience. To a certain degree, the cross-cultural care encounter between nurses and patients is not necessarily an encounter, but an intercultural experience gradually formed by the individual's own continuous learning and social practice under a dual role of environment and education.

### 4.4. Culturally Competent Care and Cross-Cultural Care Encounter Have Deviated

This study found that the average score of the imported nurses' culturally competent care was 218 ± 31.09, and the score was at a high level ([Supplementary-material supplementary-material-1]), indicating that Lhasa has nurses with higher cultural care ability. Pearson correlation analysis was conducted on the total score of the cross-cultural care encounter, cross-cultural care activity, and each dimension. The results showed that *r* = 0.126 (*P* = 0.029) (Table 3), indicating that the higher the nurses' cultural care ability, the more cross-cultural care encounter there is, the opposite of what is expected. Previous studies in China have shown that the higher the awareness of cross-cultural care, the higher the culturally competent care of nurses is [32], but the results of foreign studies by scholars such Tavallali et al. [40] were consistent with our study when they investigated the cross-cultural care encounters of immigrant nurses caring minority patients. Peng et al. [19] studied this problem in multicultural nursing of foreign patients and proposed that although the culturally competent care of Lhasa imported nurses is at a high level, there are more cross-cultural care encounters in clinical nursing work. Purnell's cultural competency model [41] proposed that mastering cultural care awareness needs to span four processes, including unconsciousness inability, conscious but inability, conscious ability, and unconscious ability. Currently, Lhasa's nursing personnel are in the second stage; they are conscious but incapable. Although imported nurses understand the importance of culture, they did not receive specialized training on Tibetan culture. As a result of weakness on the cultural knowledge of Tibetan folk language, eating habits, social etiquette, and other cultures, nurses did not know how to apply this knowledge effectively. That might be related to the particularity of Tibetan folk culture.

To further improve the quality of nursing in Tibet, the imported nurses need to cross the above four stages achieving unconscious ability, consciously learn about the Tibetan culture, and provide care suitable for the Tibetan cultural background. Nursing familiarized with the Tibetan culture can be better accepted by patients; the patient visit rate and satisfaction rate can improve. Limited resources can be utilized to maximize the overall health level of Tibet.

### 4.5. Cross-Cultural Care Encounter Is Positively Correlated with Patients' Cultural Care Needs

There is a correlation between nurses' cross-cultural care encounter and patients' cultural care needs ([Supplementary-material supplementary-material-1], *r* = 0.183, *P* = 0.003), in which patients have a close correlation with the need to respect folk customs and eating habits. When patients realized they have cultural differences with nurses, they might pay more attention to their own culture. Therefore, the stronger the patient's demand for cultural care, the more nurses experience the cross-cultural care encounter [42]. The study found that patients have a higher proportion of cultural care needs on nurses' communication skills, privacy protection, religious beliefs, social etiquette, respect for customs, and eating habits. However, in this study, the nursing ability was at a medium level, that is, conscious and incapable, which needs improvement. The Tibetan communication ability and cultural background were the main factors affecting the cross-cultural care encounter between nurses and patients. The language barrier between nurses and patients should be paid attention carefully. It is necessary to organize relevant training, improve the daily Tibetan communication ability and cultural coordination ability of the nurses, deepen new nurses' understanding of Tibetan folk culture, improve the application of cross-cultural need theoretical knowledge, and promote the use of Tibetan language for communication.

### 4.6. Study Limitations

Constrained by lacking research and limited time, the survey only investigated the nurses and patients in tertiary hospitals in Lhasa. The research scope was limited, and the sample size was small. Also, due to the limitations of the research conditions, we only could discuss the influence of general information, sociological data, nurses' cultural care ability, and patient cultural needs on cross-cultural care encounter. Some of the tools used in this survey have only theoretical support from the literature review, and they are still in the preliminary discussion stage.

### 4.7. Implications

In recent years, with the improvement of living standards, patients are not satisfied with disease treatment; they demand more attention to humanistic care, such as their cultural care. Given the development status of cross-cultural care between nurses and patients in this study, the following observations are made: Research in this subject should expand the sample size and research scope to make the research more representative. Besides, with the humanization of medical care at the core, it is suggested to expand the research direction. We should adopt scientific research methods that are carried out simultaneously with quantitative and qualitative research, to understand further and analyze the current situation of cross-cultural care in Tibet and propose diversified cross-cultural care strategies to improve the patient care ability of nurses and the quality of care services.

## 5. Conclusions

This study found that cross-cultural care encounters of imported nurses in Lhasa are held at a high level. Different ages, Tibetan language proficiency, technical titles, participation in the Tibetan language, and humanities educational training are the main influencing factors. The score of the cross-cultural care encounter of Tibetan patients in Lhasa is at a medium level; different occupations, home areas, and Chinese language proficiency are the main influencing factors. Tibetan patients of different occupations, Chinese language proficiency, and religious beliefs have different scores of cultural care needs, which is high. Cross-cultural care encounters of imported nurses in Lhasa are positively related to the cultural care ability, which is in the second stage (conscious and incapable status). The cross-cultural care encounter of imported nursing staff is positively correlated with the patient's cultural care needs, suggesting that the higher the demand of cultural care for Tibetan patients, the more cross-cultural care encounters nurses and patients experienced.

## Figures and Tables

**Table 1:** Single-factor analysis of the cross-cultural care encounter scores of imported nurses (*n* = 300).

Variable	Grouping	Mean ± SD	*F*/*t*/*z*/*x*^2^	*P*
Age (years)	18-25	64.56 ± 13.13	5.777	0.003^∗∗^
	26-30	61.38 ± 11.63		
	>30	58.82 ± 9.80		
Gender	Male	67.59 ± 12.37	-2.106	0.036^∗^
	Female	61.38 ± 11.77	1 > 2	
Nationality	Han	61.79 ± 11.92	0.638	0.524
	Hui	58.67 ± 9.48		
Religious belief	Buddhism	58.67 ± 9.48	2.100	0.124
	Islam	56.00 ± 8.12		
	None	62.10 ± 12.02		
Educational level	Secondary school	58.64 ± 12.25	9.359^①^	0.009^∗∗^
	Junior college	59.98 ± 11.07		
	Undergraduate	63.73 ± 12.23		
Tibetan language training	Yes	55.14 ± 11.87	-4.569	0.000^∗∗^
	No	63.28 ± 11.36		
Understanding Tibetan	Yes	61.54 ± 15.62	-3.199	0.001^∗∗^
	No	61.76 ± 16.24		
Humanistic training	Yes	65.38 ± 10.96	-2.263	0.024^∗^
	No	61.05 ± 11.93		
Hospital level	Grade III Class A	61.42 ± 12.33	-0.531	0.596
	Grade III Class B	62.43 ± 11.88		
Hospital category	Comprehensive	61.93 ± 12.00	0.921	0.358
	Special	58.67 ± 9.42		
Years working in Tibet	0-5	64.71 ± 13.63	3.781	0.049^∗^
	6-10	62.75 ± 12.22		
	11-15	62.80 ± 13.18		
	>16	59.57 ± 10.83		
Departments	Internal medicine	67.97 ± 11.5	2.781	0.330
	Surgery	62.66 ± 12.75		
	Obstetrics and gynecology	66.65 ± 10.91		
	Pediatrics	60.69 ± 9.00		
	Emergency	57.25 ± 13.70		
	Operating room/ICU	68.17 ± 14.36		
Job title	Nurse	61.63 ± 11.85	-0.095	0.924
	Head nurse	62.61 ± 12.25		
Technical titles	Nurse	62.59 ± 11.41	7.432	0.024^∗^
	Senior nurse	62.18 ± 11.10		
	Supervisor nurse	60.13 ± 12.87		

Note: ^∗^<0.05 and ^∗∗^<0.01 were of statistical significance and ① was the Kruskal-Wallis test result, *H*.

**Table 2:** Multifactor analysis of the cross-cultural care encounter scores of imported nurses (*n* = 300).

Variable	*B*	Beta	*t*	*P*
Constant	49.954	**—**	11.361	0.000^∗∗^
Age	-2.264	-0.183	-3.472	0.001^∗∗^
Participation in humanistic training	-4.540	-0.168	-3.116	0.002^∗^
Technical titles	1.950	0.114	2.734	0.007^∗^
Tibetan language ability	4.258	0.146	2.247	0.016^∗^

Note: *R*^2^ = 0.255, adjusted *R*^2^ = 0.194, and *F* = 9.992.

**Table 3:** The correlation between cross-cultural care encounter scores and culturally competent care scores for imported nurses (*n* = 300).

Dimension	Cross-cultural care situation	
	*R* value	*P* value
Cultural care awareness	0.156^∗∗^	0.007
Cultural care knowledge	0.156^∗∗^	0.007
Cultural care skills	0.126^∗^	0.029
The total score of cultural care ability	0.126^∗^	0.029

Note: ^∗^*P* < 0.05 and ^∗∗^*P* < 0.01 were of statistical significance.

**Table 4:** Correlation analysis of cross-cultural care encounter scores of nurses and patients' cultural care needs.

Dimension	Cross-cultural care of nurses	
	*R* value	*P* value
Total score of patients' cultural care needs	0.183^∗∗^	0.003
Privacy requirements	0.067	0.258
Ward in Tibetan style	0.082	0.193
Respecting folk customs	0.127^∗^	0.043
Social etiquette requirements	0.108	0.085
Respecting eating habits	0.236^∗∗^	0.000
Tibetan language communication needs	0.008	0.900
Respecting religious beliefs	0.084	0.181

Note: ^∗^*P* < 0.05 and ^∗∗^*P* < 0.01 were of statistical significance.

## Data Availability

The research article data used to support the findings of this study are included with in the article.

## Supplementary Materials

Supplementary Materials Supplement 1: general Tibetan patient demographics (*n* = 255). Supplement 2: general nurse demographics (*n* = 300). Supplement 3: the cross-cultural care need scores of Tibetan patients (*n* = 255). Supplement 4: cross-cultural care ability scores for imported nurses (point). Supplement 5: cross-cultural care encounter scores for imported nurses (points).

## References

[1] Dong B. Y. (2017). “Internet +”era to explore a new model of Tibet's medical poverty alleviation service. *Chinese Journal of Management in Chinese Medicine*.

[2] Li G. D. (2018). Research on the Uneven Distribution of Medical Resources in Tibet. *Tibet Science and Technology*.

[3] Zhao Y. Z. (2019). Analysis of the role of telemedicine in promoting the development of health in Tibet. *World Latest Medicine Information*.

[4] Xu P. H. (2019). Group-type aid to help build a new era of healthy Tibet construction. *China Health*.

[5] Zhaxi, Xu P. H. (2019). Da-Wa strive to build a healthy Tibet, serve the people of all ethnic groups in the plateau - review of the development of medical and health care in Tibet. *Modern Preventive Medicine*.

[6] Takman C. A., Severinsson E. I. (1999). A description of health care professionals’ experiences of encounters with patients in clinical settings. *Journal of Advanced Nursing*.

[7] Holopainen G., Kasen A., Nystrom L. (2014). The space of togetherness – a caring encounter. *Scandinavian Journal of Caring Sciences*.

[8] Tavallali A. G., Jirwe M., Kabir Z. N. (2017). Cross-cultural care encounters in paediatric care: minority ethnic parents’ experiences. *Scandinavian Journal of Caring Sciences*.

[9] Jirwe M., Gerrish K., Emami A. (2010). Student nurses’ experiences of communication in cross-cultural care encounters. *Scandinavian Journal of Caring Sciences*.

[10] Dan J. Z. (2001). Care of patients with Tibetan cultural background. *Tibetan Medical Journal*.

[11] Bai M., Bao J. N., Jiang R. X., Cao X. B., Li F. (2017). Analysis of the intercultural care encounter of Tibetan nursing talents. *Journal of Nursing*.

[12] Song L. Y. (2010). Value and significance of multicultural nursing. *Journal of Nursing Administration*.

[13] Campinha-Bacote J. (2002). The process of cultural competence in the delivery of healthcare services: a model of care. *Journal of Transcultural Nursing*.

[14] Carroll J., Epstein R., Fiscella K., Gipson T., Volpe E., Jean-Pierre P. (2007). Caring for Somali women: Implications for clinician–patient communication. *Patient Education and Counseling*.

[15] Kai J., Beavan J., Faull C. (2011). Challenges of mediated communication, disclosure and patient autonomy in cross-cultural cancer care. *British Journal of Cancer*.

[16] Lindsay S., King G., Klassen A. F., Esses V., Stachel M. (2012). Working with immigrant families raising a child with a disability: challenges and recommendations for healthcare and community service providers. *Disability and Rehabilitation*.

[17] Betancourt J. R., Green A. R., Carrillo J. E. (2000). The challenges of cross-cultural healthcare – diversity, ethics, and the medical encounter. *Bioethics Forum*.

[18] Lee K. E. (2018). Effects of team-based learning on the core competencies of nursing students: a quasi-experimental study. *Journal of Nursing Research*.

[19] Peng Y. Q., Yu H. P., Zhang X. L. (2015). The application of problem management in intercultural nursing of foreign patients. *Journal of Nursing Research*.

[20] Baohua M. (2013). *Survey and analysis on nursing needs and degree of satisfication of foreign patients in Shaoxing Country area*.

[21] Yan J., Shen N., Yan R. Q. (2004). Current situation of human resources and suggestions for improvement of nursing specialty in China. *Journal of Nursing Administration*.

[22] Nicholas D. B., Hendson L., Reis M. D. (2014). Connection versus disconnection: examining culturally competent care in the neonatal intensive care unit. *Social Work in Health Care*.

[23] Shi Y. F. (2013). The population development and characteristics of Tibet from the sixth census data. *Journal of Tibet University of Nationalities (Philosophy and Social Sciences Edition)*.

[24] Liu S. H. (2017). Investigation and analysis of nursing satisfaction of inpatients in military hospital of Tibet plateau. *Tibet Science and Technology*.

[25] Hu L. Y. (2011). *Investigation and countermeasure research of multi-culture nursing cognition of clinical nurses*.

[26] Peng Y. Q. (2009). *Cognitive investigation and intervention research on multicultural nursing of nursing interns*.

[27] Song X. S., Peng Y. Q. (2015). Study on the status quo and influencing factors of Shanghai community nurses' cultural care ability. *Surgery*.

[28] Huang Y. L., Yates P., Prior D. (2009). Factors influencing oncology nurses' approaches to accommodating cultural needs in palliative care. *Journal of Clinical Nursing*.

[29] Weber O., Sulstarova B., Singy P. (2016). Cross-cultural communication in oncology: challenges and training interests. *Oncology Nursing Forum*.

[30] Palmer N. R., Kent E. E., Forsythe L. P. (2014). Racial and ethnic disparities in patient-provider communication, quality-of-care ratings, and patient activation among long-term cancer survivors. *Journal of Clinical Oncology*.

[31] Üzar-Özçetin Y. S., Tee S., Kargın M. (2020). Achieving culturally competent cancer care: a qualitative study drawing on the perspectives of cancer survivors and oncology nurses. *European Journal of Oncology Nursing*.

[32] Zhou Y., Li C. Y., Zhou D. B. (2004). The absence of multicultural care and the response to care education. *Jilin Medical*.

[33] Zhang Y. Y., Jiang A. L. (2013). Development and thinking of intercultural nursing in China. *Nursing Research*.

[34] Griswold K., Zayas L. E., Kernan J. B., Wagner C. M. (2007). Cultural awareness through medical student and refugee patient encounters. *Journal of Immigrant and Minority Health*.

[35] Peng Y. Q., Yao M. L., Zhang L. P. (2009). Investigation and analysis of multicultural nursing needs of foreign patients in pudong new area. *PLA Nursing Journal*.

[36] Kang Y. (2007). Problems and countermeasures in multicultural nursing that nurses easily neglect. *Journal of Nursing (Surgical Version)*.

[37] Pu X. J., Chen H. (2014). Perioperative care of liver hydatid in Tibetan patients. *Gansu Medicine*.

[38] Nageswara Rao A., Warad D., Rodriguez V. (2017). Cross-cultural care training for pediatric hematology/oncology fellows. *MedEdPORTAL Publications*.

[39] Balzora S., Abiri B., Wang X. J. (2015). Assessing cultural competency skills in gastroenterology fellowship training. *World Journal of Gastroenterology*.

[40] Tavallali A. G., Kabir Z. N., Jirwe M. (2014). Ethnic Swedish parents' experiences of minority ethnic nurses' cultural competence in Swedish paediatric care. *Scandinavian Journal of Caring Sciences*.

[41] Purnell L. (2002). The Purnell model for cultural competence. *Journal of Transcultural Nursing*.

[42] Rydström I., Dalheim Englund A. C. (2015). Meeting Swedish health care system: immigrant parents of children with asthma narrate. *Clinical Nursing Research*.

