# Supplementary material for: The Challenge of Cross-Cultural Care Encounters: Perspective of Imported Nurses in Lhasa, Tibet
Source: Biomed Res Int. 2020 Apr 8;2020:3159178. doi: 10.1155/2020/3159178 (PMC7171642; doi:10.1155/2020/3159178)
Supplement: Supplementary Materials — Supplement 1: general Tibetan patient demographics (n = 255). Supplement 2: general nurse demographics (n = 300). Supplement 3: the cross-cultural care need scores of Tibetan patients (n = 255). Supplement 4: cross-cultural care ability scores for imported nurses (point). Supplement 5: cross-cultural care encounter scores for imported nurses (points). [file 3159178.f1.docx]

**Supplement 1. General Tibetan patient demographics (n=255)**

| **Item** | **Classification** | **Case number (n)** | **Constituent ratio (%)** |
| --- | --- | --- | --- |
| Age (years) | ＜20 | 56 | 22.0 |
|  | 21-29 | 68 | 26.7 |
|  | 30-39 | 38 | 14.9 |
|  | 40-49 | 51 | 20.0 |
|  | ＞50 | 42 | 16.5 |
| Gender | Male | 110 | 43.4 |
|  | Female | 145 | 56.6 |
| Religion | Yes | 247 | 96.9 |
|  | No | 8 | 3.1 |
| Language Proficiency | Understand Chinese | 65 | 37.3 |
|  | Do not understand Chinese | 190 | 62.7 |
| Educational level | Primary school and below | 105 | 41.2 |
|  | Junior high school | 37 | 14.5 |
|  | Senior high school/vocational high school/technical secondary school | 54 | 21.2 |
|  | College degree and above | 59 | 23.1 |
| Occupation type | Farmers and herdsmen | 110 | 43.2 |
|  | Student | 60 | 23.5 |
|  | Buddhist monks and nuns | 8 | 3.1 |
|  | Worker | 44 | 17.3 |
|  | Cadre | 33 | 12.9 |
| Home location | Countryside | 146 | 57.3 |
|  | Cities and towns | 109 | 42.8 |
| Marital status | Unmarried | 80 | 31.4 |
|  | Married | 172 | 68.2 |
|  | Divorce/widowed | 3 | 0.4 |

**Supplement 2. General nurse demographics (n=300)**

| **Item** | **Grouping** | **Number (n)** | **Constituent ratio (%)** |
| --- | --- | --- | --- |
| Gender | Male | 16 | 5.3 |
|  | Female | 284 | 94.7 |
| Age (years) | 18-25 | 103 | 34.3 |
|  | 26-30 | 110 | 36.7 |
|  | ＞31 | 87 | 29.0 |
| Marital status | Unmarried | 90 | 30.0 |
|  | Married | 199 | 66.3 |
|  | Divorced | 11 | 3.7 |
| Ethnicity | Han | 294 | 98.0 |
|  | Hui | 6 | 2.0 |
| Religious beliefs | Buddhism | 15 | 5.0 |
|  | Islam | 6 | 2.0 |
|  | None | 279 | 93.0 |
| Educational level | Undergraduate | 148 | 49.3 |
|  | Junior college | 130 | 43.3 |
|  | Secondary school | 22 | 7.3 |
| Years working in Tibet | 0-5 | 119 | 39.7 |
|  | 6-10 | 108 | 36.0 |
|  | 11-15 | 41 | 13.7 |
|  | ＞15 | 32 | 10.6 |
| Tibetan language training | Yes | 57 | 19.0 |
|  | No | 243 | 81.0 |
| Understand Tibetan | Yes | 39 | 13.0 |
|  | No | 261 | 87.0 |
| Humanistic training | Yes | 47 | 15.7 |
|  | No | 253 | 84.3 |
| Hospital level | Grade A class 3 | 206 | 68.7 |
|  | Grade B class 3 | 94 | 31.3 |
| Hospital category | Comprehensive | 282 | 94.0 |
|  | Specialized | 18 | 6.0 |
| Medical department | Pediatrics | 55 | 18.3 |
|  | Obstetrics and Gynecology | 26 | 8.7 |
|  | Internal medicine | 55 | 18.3 |
|  | Surgery | 148 | 49.3 |
|  | ICU | 6 | 2.0 |
|  | Emergency | 4 | 1.3 |
| Job title | Nurse | 269 | 89.7 |
|  | Head nurse | 31 | 10.3 |
| Technical titles | Nurse | 98 | 32.7 |
|  | Senior nurse | 158 | 52.7 |
|  | Supervisor nurse | 44 | 14.7 |

**Supplement 3. The cross-cultural care needs scores of Tibetan patients (n=255)**

| **Dimension** | **Item** | **Total score** | **Average score** |
| --- | --- | --- | --- |
| Tibetan language communication | 3 | 7.81±2.250 | 3.90±0.22 |
| Privacy requirements | 3 | 11.62±2.45 | 3.87±0.25 |
| Respecting eating habits | 3 | 11.45±1.623 | 3.81±0.11 |
| Respecting customs | 3 | 7.13±1.090 | 3.57±0.10 |
| Respecting religious beliefs | 3 | 10.55±2.162 | 3.51±0.14 |
| Social etiquettes | 3 | 6.41±1.827 | 3.21±0.18 |
| Ward in Tibetan style | 2 | 4.63±1.972 | 2.31±0.20 |
| Total score | 20 | 60.56±13.73 | 3.03±0.80 |

**Supplement 4. Cross-cultural care ability scores for imported nurses (point, $\mathop{x}\limits^{-} \pm s$)**

| **Dimension** | **Item** | **Total score** | **Average item score** |
| --- | --- | --- | --- |
| Awareness of cultural care | 16 | 58.52±9.31 | 3.65±0.58 |
| Knowledge of cultural care | 16 | 56.32±9.08 | 3.52±0.57 |
| Skills of cultural care | 28 | 93.74±8.57 | 3.34±0.38 |
| Total score | 60 | 208.58±25.09 | 3.47±0.24 |

**Supplement 5. Cross-cultural care encounter scores for imported nurses (points,$\mathop{x}\limits^{-} \pm s$)**

| **Item** | **Item number** | **Total score** | **Average score** |
| --- | --- | --- | --- |
| Language communication | 4 | 15.17±3.41 | 3.80±0.86 |
| Dietary habits | 2 | 7.14±1.80 | 3.57±0.90 |
| Different religious beliefs | 2 | 7.10±1.82 | 3.55±0.91 |
| Different folk customs and etiquette | 2 | 6.24±1.59 | 3.12±0.80 |
| Different health-related social knowledge | 2 | 6.15±1.42 | 3.08±0.71 |
| Different ward environments | 2 | 5.14±1.73 | 2.57±0.87 |
| Different folk taboos | 3 | 8.35±2.51 | 2.78±0.84 |
| Ethnic discrimination and prejudice | 3 | 6.49±1.52 | 2.16±0.51 |
| Total score | 20 | 61.73±11.86 | 3.09±0.59 |
